# A systematic review: Are herbal and homeopathic remedies used during pregnancy safe?

**DOI:** 10.4102/curationis.v39i1.1514

**Published:** 2016-04-13

**Authors:** Haaritha Boltman-Binkowski

**Affiliations:** 1 Advanced Midwifery and Neonatal Nursing, University of the Western Cape, South Africa

## Abstract

**Background:**

Herbal and homeopathic remedies have been used to assist with childbearing and pregnancy for centuries. Allopathic (‘Western’) medicine is traditionally avoided during pregnancy because of limited drug trials and the suspected teratogenic effects of these medications. This has led to an increase in the use of herbal and homeopathic remedies, as they are viewed to have no teratogenic effect on the developing foetus. Health providers are faced with questions from their clients regarding the safety of these remedies, but much of the evidence about these herbal and homeopathic remedies is anecdotal and few remedies have been tested scientifically.

**Objectives:**

By conducting a systematic review, the primary objective was to evaluate maternal and neonatal outcomes of ingested herbal and homeopathic remedies during pregnancy.

**Method:**

A systematic review was conducted to synthesise all the evidence with the purpose of evaluating the safety of herbal and homeopathic remedies based on adverse maternal and neonatal outcomes. Only randomised and quasi-randomised controlled trials that met all inclusion criteria were included in the review.

**Results:**

The ingestion of ginger for nausea and vomiting during pregnancy was shown to have no harmful maternal or neonatal effects. Ingestion of castor oil for induction of labour showed a tendency towards an increase in the incidence of caesarean section and meconium-stained liquor, warranting further research into its safety issues.

**Conclusion:**

Larger randomised controlled trials need to be conducted, especially in South Africa, to establish the safety and efficacy of commonly-used remedies.

## Introduction

Herbal and homeopathic remedies have been used to assist with childbearing and pregnancy for centuries (Lee 1999). Complementary and alternative medicines (CAMs) have been used in various ways to alleviate common pregnancy ailments, to ease the labour process and to assist with recovery after childbirth. Most of the evidence supporting these remedies is anecdotal, or passed down to younger generations by rich cultural oral traditions. Health providers, especially nurses and midwives, are continually faced with the remedies clients use and require scientific information in order to present evidence-based care to clients.

### Problem statement

Literature poorly documents the use and effects of CAMs in South Africa, although the use of a traditional Zulu remedy, *impila* (*Callilepis laureola*) was banned as recently as the 1980s in KwaZulu-Natal because of its reported toxicity (Varga & Veale 1997). A recent study on the *Callilepis laureola* plant revealed hepatotoxic effects, especially in children (Stewart *et al*. 2002). It is also common knowledge that users of herbal products assume that they are safe for use during pregnancy because they are ‘natural’; these users will revert to the use of herbal products, especially during pregnancy and breastfeeding when the use of most evidence-based medication is contra-indicated. Therefore, the need has arisen to amalgamate evidence about the use of these traditional remedies in order to provide midwives with research evidence to present to clients, with the aim of enabling them to make an informed decision.

#### Aims of the study

The aims of the study were to review the evidence from randomised controlled trials that tested the effects of herbal and homeopathic remedies that women were using during pregnancy and labour and to report on their adverse maternal and neonatal outcomes.

#### Background

The use of herbs and other natural substances as therapeutic medications has been handed down and documented by various ancient civilisations and these continue to be used today. The ancient Egyptians specifically described the uses of crocodile dung, honey and sour milk for contraception; as well as detailing the anatomy and ailments of the female reproductive system (Aboelsoud 2010). In 2005, a systematic review found that herbal and homeopathic remedies, both in ingested and topical forms, were used internationally to treat common ailments during pregnancy (Anderson & Johnson 2005). Substances documented in their review ranged from ingested ginger for prenatal nausea to the application of moxibustion to treat breech presentation; some of the remedies were found to be effective (Anderson & Johnson 2005).

Recently, herbal remedies are most often used for treating the most common pregnancy-related problems, such as nausea, stretch marks and varicose veins. They have also been advocated to shorten or, where appropriate, increase the duration of the gestational period, augment or induce labour, decrease the duration of the birthing process, relieve perineal pain after birth, alleviate pain associated with cracked nipples and engorged breasts and increase breast milk production (Ernst 2002; Olson 2001). More commonly, herbal and homeopathic remedies will be used by women who are not able to access healthcare facilities.

Very few compounds have been tested scientifically for their active ingredient, their mechanism of action and any adverse effects demonstrated during pregnancy, birth and breastfeeding. Practising midwives traditionally use a variety of herbs and nutritional supplements during the labouring process. Unfortunately, much of this information is anecdotal and has very little scientific support, making it difficult to evaluate safety (Olson 2001). In addition, concerns have been raised about the adverse effects of these remedies. Many reports of adverse effects are published as case studies and not as the results of a controlled trial (Anderson & Johnson 2005). Reported adverse effects of the ingestion of *isihlambezo* (a general pregnancy health tonic used by Zulu women), for example, include: meconium staining of amniotic fluid; increased rate of caesarean section; and the possibility of acute renal failure (Ernst 2002; Mabina, Pitsoe & Moodley 1997). The following side effects have also been reported in the neonate: ascites of various degrees and hepatomegaly; mental retardation; cardiac complications; and enlarged genitalia (Ernst 2002; Mabina *et al*. 1997). It is evident that although use of herbs during pregnancy is well documented, safety issues about their use need to be explored.

#### Research objectives

The primary objective of the study was to ascertain the safety of herbal and homeopathic remedies that women were using during the pre-, intra- and postpartum periods. This was done by conducting a meta-analytic systematic review.

#### Contribution to the field

The use of herbs and homeopathy during the ante-, intra- and postpartum period is well-documented; however, much of the evidence presented is anecdotal. Few randomised trials about the use of these remedies have been conducted. There are no systematic reviews that combine the results of the randomised controlled trials in order to present the evidence about the safety of the use of herbal remedies.

There have been reported adverse effects of herbal use for both mother and neonate scattered across various studies, with certain remedies being banned from use in South Africa because of reported toxicity (Varga & Veale 1997). Reliance on traditional herbal remedies has been associated with poor antenatal clinical care, coping with the stress of urbanisation and cultural transitioning (Varga & Veale 1997).

Midwives are the first point of contact for many expectant mothers in South Africa. This article could significantly contribute to the knowledge base about the safety of the use of herbal and homeopathic remedies in relation to pregnancy. When midwives utilise the evidence-based knowledge in this article, it could ensure that there is a shared decision-making model of care. This is empowering for clients and opens communication on a cultural level, whilst ensuring safe and effective midwifery care.

### Definition of key concepts

#### Adverse effect

**O**pposite or antagonistic effect, a negative effect (Reader’s Digest Universal Dictionary 1988:32b).

#### Allopathy/allopathic

A system of medical treatment or therapy whereby an environment to treat disease/abnormal conditions is created, which is hostile to the disease itself, for example, antibiotics to treat infections. This therapeutic system is used to describe modern medical practices and includes both medication and surgical techniques (Mosby’s Medical Dictionary 2009).

#### Complementary and alternative medicine

Therapeutic practices and medications which are allied to the biomedical model of medicine, for example, reflexology, acupuncture, homeopathy, herbology and iridology (Reader’s Digest Universal Dictionary 1988:326c).

#### Efficacy

The ability of a drug to produce an effect (intended or otherwise), regardless of the dose administered (Mosby’s Medical Dictionary 2009).

#### Herbal

In this study, herbal medication/remedy refers to a system of treatment and prevention of diseases through administering botanical therapies to treat/prevent disease conditions. These botanical therapies may be administered in various forms – in their natural form, teas, tinctures, drops, ointments, vapours, or as pill or capsule form preparations.

#### Homeopathy

A therapeutic system designed in the late 18th century by Dr Samuel Hahnemann. The system is based on the belief that ingesting small amounts of a drug which causes a disease (or a drug which simulates symptoms of a disease) may actually stimulate the body to cure itself – ‘like cures like’ (Mosby’s Medical Dictionary 2009).

#### Meta-analyses

This is a quantitative research method that utilises specific statistical techniques to amalgamate the results from different studies to obtain the overall effect of a particular intervention on a defined outcome (Cook, Mulrow & Haynes 1997).

#### Randomised controlled trial

A trial designed to test the effect of a specific intervention. The trial design requires participants to be relatively similar, two test group – one which is exposed to the invention and the other not. Both groups should be allocated to either treatment or control entirely randomly. In this way, it can be certain that the effect is a result of the intervention and not because of other variables.

#### Safety

In this study, safety of an intervention is one that is defined by producing no adverse events to mothers or neonates after being exposed to the intervention.

## Research design

The systematic review method is a method of quantitative research that was used to obtain the results presented in this study. Scientific systematic reviews are used to collate studies that address the same topic, explaining differences and similarities amongst the studies. The application of scientific strategies is used to limit bias and to amalgamate all relevant studies that address a specific clinical question. Specific statistical methods, such as the meta-analysis of outcomes, are used to combine and summarise the results of the primary trials, as well as to limit bias and random error. Scientific systematic reviews may strengthen the link between effective evidence and clinical practice. They are also increasingly used to inform medical decision making, establish clinical guidelines and to plan future research studies (Cook *et al*. 1997).

A systematic review was conducted by means of a quantitative meta-analysis to address the following review question: *How safe is the ingestion of herbal and homeopathic remedies during the pre-, intra- and postpartum period?*

### Research approach and method

#### Search strategy

An extensive electronic search was carried out to ensure that all relevant literature was included in the review. Key words for the search included: herbs; pregnancy; homeopathy; breastfeeding; antenatal; intrapartum; birth; labour; postpartum care; puerperium; induction of labour; alternative remedies; childbearing; traditional midwifery; randomised (all variations); and trial. A comprehensive list of names of herbs was also included in the search. Key words were used in isolation but also in combination with one another. Those terms were physically entered into the electronic biomedical databases. Databases used were PubMed, EBSCO, Medline, CINAHL, Science Direct and the Cochrane Controlled Trials register. In addition, Google Scholar was checked for relevant references (by using the same search terms) to discover articles that might not have reflected on any of the databases. Reference lists of articles were checked for further trials; however, a hand search of library journals was not conducted, since electronic data included the journals in print.

#### Selection of studies

Studies were selected for review on the basis that they had tested the effects of ingested herbal and homeopathic remedies during pregnancy and labour – whether they reported adverse maternal and/or neonatal outcomes – and met the inclusion and exclusion criteria outlined below. Exclusion criteria for data or methodology included an inappropriate format for inclusion in the review, articles with data that had not been published at the time of the review (the years 2000–2014) and articles that were not printed in the English language. Data were considered inappropriate for inclusion when it was qualitative in nature, anecdotal, non-experimental, or when it was determined that an independent variable was manipulated as determined by the quality assessment of each article. Articles were also excluded when they did not report on an outcome that other studies also presented. Methodological issues for exclusion were established whether the study presented insufficient evidence with unreliable results, whether conclusions were not sufficiently based on scientific evidence, or whether the study presented questionable validity. All included articles were assessed for quality by both reviewers, using the Johns Hopkins Nursing Evidence-Based Practice Research Evidence Appraisal tool (Newhouse *et al*. 2007).

Studies were included in the review based on the PICOS (participant, intervention, comparison, outcomes and studies) principles outlined below.

**Participants:** The participants included in the review were studies that included women of reproductive age (21–51 years), who were either pregnant at the time of the original study, or who had already borne children and who had used herbal or homeopathic remedies during the pregnancy and labour period to alleviate common pregnancy-related illnesses. Common ailments include, but are not limited to, nausea and vomiting during pregnancy and induction of labour.

**Interventions and comparisons:** The types of interventions considered for inclusion in review were various herbal and homeopathic remedies that were commonly ingested by women as an intervention during pregnancy and labour. Comparisons that were considered for inclusion were ingested herbal or homeopathic remedies versus placebos for nausea and vomiting during pregnancy, as well as ingested herbal or homeopathic remedies versus placebos for induction of labour.

**Outcomes:** Maternal adverse events were defined as an increase in the incidence of caesarean section and/or incidence of spontaneous abortion. Neonatal adverse events included incidence of congenital abnormalities, still births and incidence of meconium-stained liquor.

**Studies:** Studies were only included in this review when they were appropriately designed to evaluate the outcome measures relevant to the safety of the use of herbal and homeopathic remedies during pregnancy and labour, defined by maternal and neonatal adverse events. The types of studies included in the review were limited to clinical trials that compared any ingested homeopathic or herbal remedies with placebo or another method for the prevention or treatment of ailments or interventions during pregnancy and childbirth. Clinical trials included in the review considered treatments versus placebo for nausea and vomiting, as well as induction of labour.

### Data extraction

**FIGURE 1:**
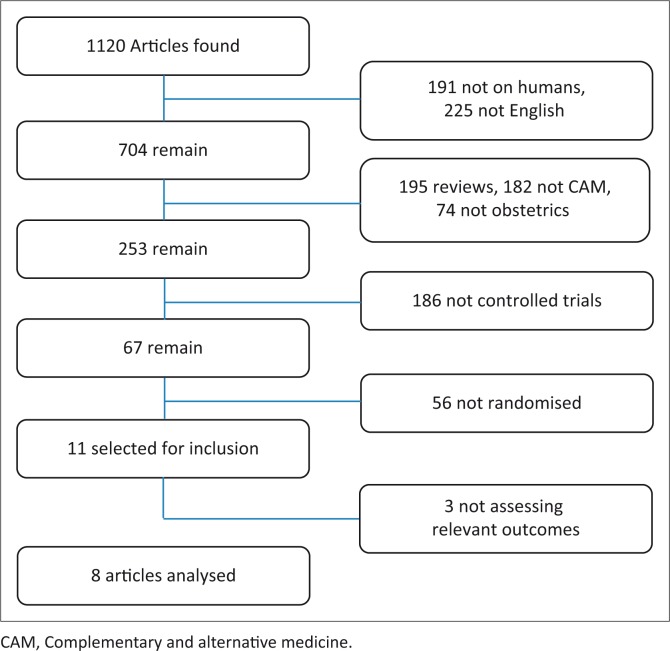
Search strategy for articles.

The abstracts of articles were consulted to ensure that all the relevant trials were included in the review. Full articles were either obtained online, when available, or ordered through the library facilities of the University of the Western Cape. The study selection was undertaken independently by both reviewers and the trials were included only when they met the inclusion criteria (Figure 1). The studies were discussed with an independent reviewer and discrepancies for inclusion (Table 1) or exclusion (Table 2) were resolved. All studies that met the broader inclusion criteria were then individually evaluated for methodological quality based on the criteria set out in the Johns Hopkins Nursing Evidence-Based Practice Research Evidence Appraisal tool by both reviewers (Newhouse *et al*. 2007). These criteria included evaluation of the strength and quality of the evidence. Strength measures ranged from level I (strong, e.g. randomised control trials) to level III, which included non-experimental, qualitative studies. The quality of the evidence was evaluated from A (high) to C (low, with major methodological flaws) and was based on the quality of results, sample size, control measures and the scientific basis for conclusions and recommendations. The data from each publication had been extracted independently by both reviewers before it was compared. There were no discrepancies in data extraction. Authors’ names, study site, intervention and trial results were not masked. The data were then entered on a special data collection sheet; one for each included study. The outcome measures of herbal use during pregnancy versus the control group were indicated on each data collection sheet.

**TABLE 1:** Characteristics of included studies.

Study	Methods	Participants	Intervention	Outcomes	Quality score
Azhari *et al*. 2006	Randomised controlled trial	Primi and multigravid, pregnant females, age 19–35 years, gestational age 40–42 weeks, singleton pregnancies, Bishop’s score less than or equal to four, intact membranes, reactive non-stress test	Castor oil, 60 mL, orally	Initiation of labour with onset of three strong uterine contractions	I B
Ensiyeh & Sakineh 2009	Double-blind randomised controlled trial	Primi and multigravid, pregnant females, aged 20–30, at 17 weeks of gestation or less who experienced nausea with or without vomiting	Ginger, 1 g/day for 4 days, orally	Changes in severity of nausea	I A
Garry *et al*. 2000	Controlled trial	Mean age of 24.5, at 40–42 weeks’ gestation, Bishop’s score of 4 or less, no evidence of uterine contractions on tocometry	Castor oil, 60 mL, orally	Onset of labour in 24 hours; 1 or more contractions every 5 minutes with cervical dilation of 4 cm or more	II B
Gilad *et al*. 2012	Randomised, double-blind, placebo-controlled trial	Singleton pregnancy, 40–42 weeks, Bishop’s score less than or equal to 7, no uterine activity and no previous caesarean section	Castor oil, 60 mL, orally	Spontaneous onset of labour within 12 hours	I A
Keating & Chez 2002	Double-blind randomised controlled trial	Primi and multigravid, pregnant females, aged 24–37 years in the first trimester (7–11 weeks of gestation), experiencing nausea and/or without vomiting, and were not taking a prescribed or over the counter antiemetic	Ginger syrup 250 mg ginger (1 tablespoon), 4× daily orally	Level of nausea	I B
Smith *et al*. 2004	Randomised, controlled equivalence trial	Women with nausea or vomiting, between 8 and 16 weeks pregnant, with dates confirmed by ultrasound	Ginger; 1 capsule of ginger (350 mg), orally	Nausea	II B
Vutyavanich, Kraisarin & Ruangsri 2001	Randomised, double-masked, placebo-controlled trial	Women with nausea of pregnancy, with or without vomiting, at or before 17 weeks gestation	Ginger; 1 g in 250 mg capsule, orally	Improvement in nausea symptoms	I B
Willetts, Ekangaki & Eden 2003	Double-blind randomised placebo-controlled trial	Pregnant women aged 22–43 years, less than 20 weeks pregnant, had experienced morning sickness daily for at least a week which had failed to respond to dietary measures	125 mg ginger extract (equivalent to 1.5 g of dried ginger) orally	Nausea	I A

**TABLE 2:** Excluded studies.

Study identifier	Reason for exclusion
Oberbaum *et al*. 2005	Study only looks at the effect of homeopathy on mild post-partum bleeding, outcome inappropriate for inclusion in this review.
Simpson *et al*. 2001	Study only looking at the duration of pregnancy, outcome inappropriate for inclusion in this review.
Ingram 2003	No maternal or neonatal adverse events reported on – no outcomes available, hence not included in this review.

### Data treatment

After the data had been extracted from the studies and entered on the data collection sheet, it was entered for analysis into the Review Manager (RevMan) Program Version 5.3 (Cochrane Reviews 13 June 2014), a specialised program designed for calculation of statistics utilised in meta-analyses. Two reviewers extracted the data and compared the data. There were no differences between the two reviewers’ extractions. A confidence interval of 95% and random relative risk were used for this review. The Mantel-Haenszel test was applied to all outcomes to ensure that repeated ‘tests of independence’ of the data were analysed.

## Results

As a result of the intensive electronic search, 1120 abstracts were found (Figure 1). A total of 416 abstracts were excluded: 191 of the excluded abstracts did not include human participants and 225 were not in the English language. A further 451 abstracts were excluded: 195 of them were not reviews, 182 were not related to CAMs and 74 did not discuss pregnancy, birth or the postnatal period. Abstracts that presented controlled trials were excluded (*n* = 186), as were 56 non-randomised controlled trials. At that stage, full-text articles were obtained and a further three articles were excluded, since they did not have relevant outcomes for synthesising into this review. A final total of eight articles met the eligible criteria and were found to be appropriate for inclusion in this review.

Two distinct categories of comparisons were analysed: ingested remedies for nausea and vomiting; and ingested remedies for induction of labour. Ginger was the only remedy used to treat nausea and vomiting during pregnancy and only castor oil was used to induce labour.

### Ingested remedies for nausea and vomiting

**FIGURE 2:**
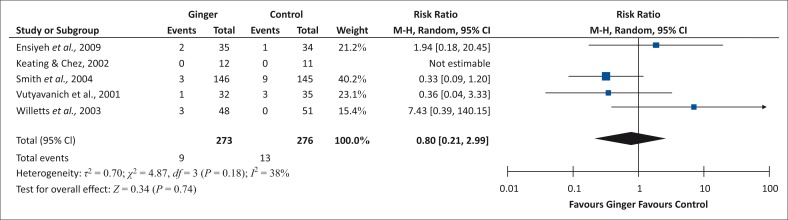
Incidence of caesarean section for ingestion of ginger.

Ginger has no significant effect on increasing the incidence of caesarean section in this study. Ingested ginger does not cause increased caesarean sections as compared to a population that does not ingest any remedies (Figure 2).

**FIGURE 3:**
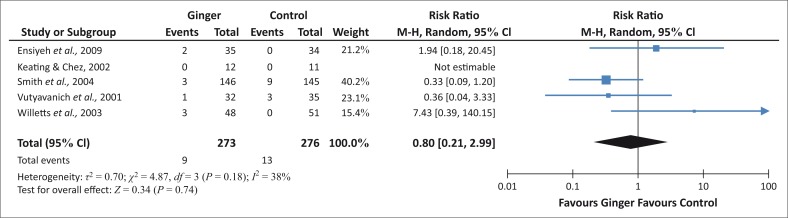
Incidence of spontaneous abortion.

For the outcome of spontaneous abortion, ingested ginger favours neither treatment nor control, so it can be concluded that ginger does not cause a significant increase in spontaneous abortion as compared to a control group (Figure 3).

**FIGURE 4:**
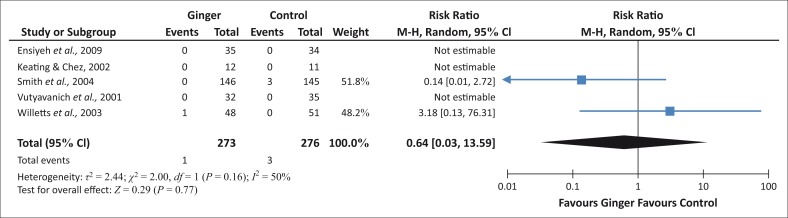
Incidence of stillbirth.

Ingested ginger fails to achieve a customary level of statistical significance for stillbirth (Figure 4). This implies that ingested ginger does not lead to a higher rate of stillbirths as compared to a general population.

**FIGURE 5:**
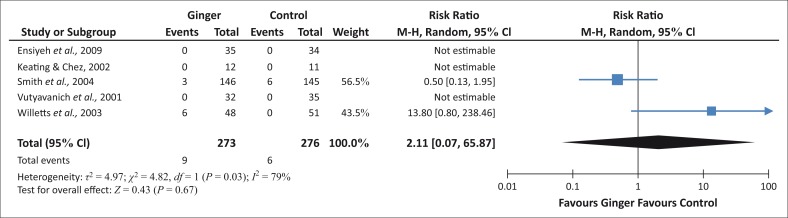
Congenital abnormalities.

Pregnant mothers who avoid ingesting ginger did not significantly experience more congenital abnormalities than those mothers in the ingested ginger group (Figure 5). Congenital abnormalities occurred in both groups, but not at levels that are unusual for the general population.

### Ingested remedies for induction of labour

**FIGURE 6:**
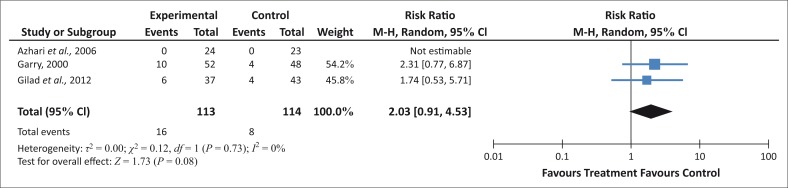
Incidence of caesarean section for ingestion of castor oil.

More women who have ingested castor oil for induction of labour, receive caesarean sections, so it is possible that ingesting castor oil might lead to a higher incidence of caesarean sections (Figure 6).

**FIGURE 7:**
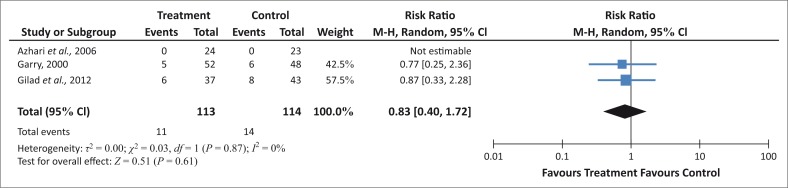
Incidence of meconium-stained amniotic fluid.

There is a tendency that ingested castor oil might have a negative effect in the neonate, since more women in the group who have ingested castor oil appear to have meconium-stained liquor than in the control group (Figure 7).

## Ethical considerations

Ethical clearance was sought from and granted by the University of the Western Cape Faculty of Community and Health Sciences Higher Degrees Committee and Senate. Patient consent was not required in a systematic review of literature.

## Discussion

### Outline of the results

Sample sizes for the studies in this review were small and only eight studies were used. A clear conclusion about safety of ingested herbal remedies cannot be formulated from this review. We can, however, conclude that ginger has no effect on incidence of caesarean sections, stillbirths, congenital abnormalities, or spontaneous abortion, which corresponds with the findings of a randomised controlled trial on ingested ginger use (Fischer-Rasmussen *et al*. 1991). Ingested castor oil shows tendencies toward increased caesarean section and meconium-stained liquor. Caution should still be exercised before these remedies are used, because of the small number of studies reviewed.

### Practical implications

Ingested ginger has been purported to be efficacious for the treatment of nausea and vomiting during pregnancy (Portnoi *et al*. 2003). Literature does not suggest that there may be any adverse effects for either the mother or neonate. The United States Pharmacopeia reports no harmful effects of using ginger (United States Pharmacopeia 2008). In addition, no adverse events have been observed with ingested ginger in a randomised controlled study on the treatment of hyperemesis gravidarum (Fischer-Rasmussen *et al*. 1991). Ginger can safely be used to treat nausea and vomiting in pregnancy, with no significant maternal or neonatal adverse events reported in the literature or found in the results of this review.

The results of the review show that more research into the adverse effects of castor oil as an induction agent needs to be conducted, even though Garry *et al*. (2000) show it to be effective for initiating labour. Data on maternal and neonatal mortality were not presented in the Cochrane review on castor oil as an induction agent (Kelly, Kavanagh & Thomas 2004). The ingestion of castor oil has been associated with various maternal and neonatal side effects, but these associations have only been recorded in case reports. Amniotic fluid embolism was a significant condition reported in a woman who had ingested castor oil during pregnancy, but a direct cause–effect relationship between the embolism and ingestion of castor oil was not established (Steingrub *et al*. 1988). El Mauhoub *et al*. (1983) report adverse neonatal events, such as moderate growth impairment, convulsions, craniofacial dysmorphia, limb reductions and vertebral defects, as being associated with the ingestion of castor oil during pregnancy. In a prospective evaluation study by Garry *et al*. (2000), no such adverse events are reported.

Sample sizes were small and most of the literature available did not include a control group, but were rather case reports, letters or anecdotal articles about the use of different remedies. The controlled trials, as stated before, had relatively small sample sizes and, even with meta-analysis, they could not provide substantial evidence on most outcomes. It is clear from the review that larger randomised controlled trials need to be conducted, especially in areas where use of a specific herbal or homeopathic remedy is entrenched in culture or tradition. Even in developed societies where tradition does not play a strong role, the return to alternative therapies, especially during pregnancy, lends support to the motivation for larger trials on specific substances.

## Limitations of the study

The primary limitation to this study was the sample sizes that were too small to draw a substantial conclusion. Also, only English language studies were included in this review, whereas much research in this field was published in Asia and the Far East. Unfortunately, the budget of this research study did not allow for the services of a translator.

## Recommendations

Health professionals have a responsibility toward their gravid clients in advocating best clinical practices with regard to the latest evidence, as well as protecting them and their foetuses from potentially harmful treatment. Whilst the use of herbal medications is rising, this review indicates that substantial evidence to advocate or contra-indicate the use of herbal or homeopathic substances is not yet available.

In the South African context, this could mean initiating professional dialogue for specific management with registered traditional healers or other practitioners. When health professionals consult with their clients during antenatal visits, enquiries should be made into the use of herbal products to open discussions about safety and efficacy. Gravid clients with complications, such as epilepsy and cardiac conditions, should be warned about possible drug interactions with any herbal medication.

## Conclusion

Health professionals should take cognisance of the prevalence of herbal and homeopathic use amongst their clients and provide clients with enough information to make a balanced decision about whether to use these substances or not. Clinicians and researchers should be encouraged to pursue further trials in this field with the purpose of building substantial evidence in relation to the safety and efficacy of herbal/homeopathic remedies during pregnancy.
